# Comparative Study of Green and Synthetic Polymers for Enhanced Oil Recovery

**DOI:** 10.3390/polym12102429

**Published:** 2020-10-21

**Authors:** Nasiru Salahu Muhammed, Md. Bashirul Haq, Dhafer Al-Shehri, Mohammad Mizanur Rahaman, Alireza Keshavarz, S. M. Zakir Hossain

**Affiliations:** 1 Department of Petroleum Engineering, King Fahd University of Petroleum and Minerals, Dhahran 31261, Saudi Arabia; g201907810@kfupm.edu.sa (N.S.M.); alshehrida@kfupm.edu.sa (D.A.-S.); 2 Center of Research Excellence in Corrosion, King Fahd University of Petroleum and Minerals, Dhahran 31261, Saudi Arabia; mrahman@kfupm.edu.sa; 3 School of Engineering, Edith Cowan University, Joondalup, WA 6027, Australia; a.keshavarz@ecu.edu.au; 4 Department of Chemical Engineering, University of Bahrain, P.O. Box 32038 Zallaq, Bahrain; zhossain@uob.edu.bh

**Keywords:** EOR, green EOR (GEOR), hydrolyzed polyacrylamide (HPAM), xanthan gum (XG)

## Abstract

Several publications by authors in the field of petrochemical engineering have examined the use of chemically enhanced oil recovery (CEOR) technology, with a specific interest in polymer flooding. Most observations thus far in this field have been based on the application of certain chemicals and/or physical properties within this technique regarding the production of 50–60% trapped (residual) oil in a reservoir. However, there is limited information within the literature about the combined effects of this process on whole properties (physical and chemical). Accordingly, in this work, we present a clear distinction between the use of xanthan gum (XG) and hydrolyzed polyacrylamide (HPAM) as a polymer flood, serving as a background for future studies. XG and HPAM have been chosen for this study because of their wide acceptance in relation to EOR processes. To this degree, the combined effect of a polymer’s rheological properties, retention, inaccessible pore volume (PV), permeability reduction, polymer mobility, the effects of salinity and temperature, and costs are all investigated in this study. Further, the generic screening and design criteria for a polymer flood with emphasis on XG and HPAM are explained. Finally, a comparative study on the conditions for laboratory (experimental), pilot-scale, and field-scale application is presented.

## 1. Introduction

**Figure 1:**
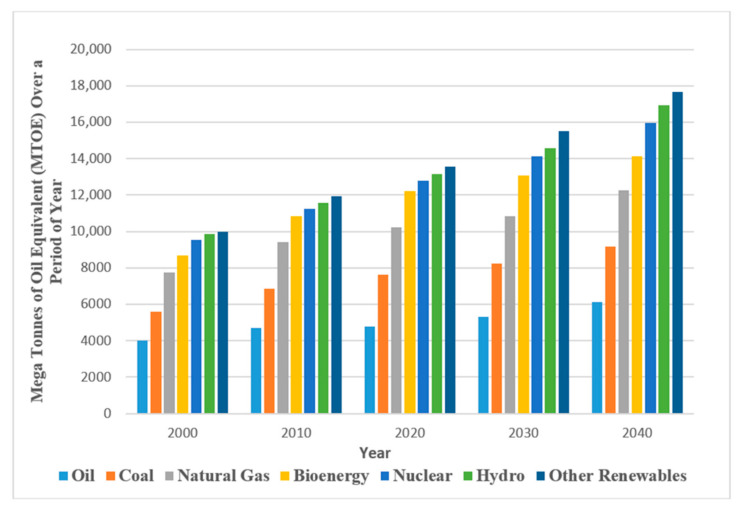
Global crude oil and energy demand. The data used were generated based on average from reference [1].

Global energy demand continues to rise as a result of industrial and life developments [1]. Currently, fossil fuels, especially oil and gas, play a vital role as compared to other sources (solar, wind, and so on) in energy production (see Figure 1). This is because alternative energy sources are yet to be able to fully satisfy world demand for energy. Therefore, it is imperative to properly harness oil reserves and maximize their production potential. Oil recovery operations, as explained by [2], have traditionally been divided into three stages: primary, secondary, and tertiary. Enhanced oil recovery (EOR) describes the employment of the tertiary recovery method when both primary and secondary techniques are rendered uneconomical, where the reservoir is yet to reach its full production potential. Chemical, gas, and thermal modes are the three conventional EOR techniques that have been applied in the recovery of typically 50–60% residual oil saturation from the original oil in place (OOIP). Although, microbial and nano-assisted technologies are another key area of interest in recent times. These types of technology (chemical, gas, and thermal) were applied from the 1960s through to the late 1980s, at a time when oil producers were looking for additional reserves in response to rising oil prices [3]. However, the process of polymer flooding as a conventional chemically enhanced oil recovery (CEOR) technology has been considered one of the most important as a result of historical field studies and applications [4]. In fact, out of 11% of CEOR global projects, 77% are attributed to polymer flood while 23% are ascribed to integrations of polymers and surfactants [1].

Polymers are chemical substances in the form of chains that are composed of long and repeated small units called monomers through a process of polymerization. To initially increase oil recovery, water flooding is completed to push the oil towards the production well. However, due to immiscibility between oil and water, no significant displacement can be achieved because of low water viscosity and heterogeneity present within a reservoir. Hence, a polymer flood can be used to annul some of these challenges. In polymer flooding, large amounts of polymer molecules that are soluble in water are injected with the injection fluid to enhance the rheological properties of the displacing fluid. Two of these include (1) increasing water viscosity for mobility control and (2) reducing residual oil saturation for better oil production [5]. Other benefits include: (1) annulling the fingering effect; (2) increasing both vertical and areal sweep efficiency; (3) maintaining low cost in comparison to other techniques [6,7,8,9,10]. Various publications by authors in this field have identified that two common (basic) polymers are used worldwide in the industry: synthetic and biopolymer types [11]. Polyacrylamides (synthetic) are manufactured by polymerization of acrylamide monomers, whereas polysaccharides (biopolymers) are commercially produced through microbial action of organisms. The main physical and chemical properties of polymers include rheological properties of shear rate, shear stress, and viscosity. However, other factors, such as the effect of salinity and temperature, retention time, permeability reduction and polymer mobility, retention, and adsorption of the polymer, are key when it comes to the study of polymers.

The addition of polymer to water alters the rheological behaviour of the water itself. This makes the solution behave as a non-Newtonian fluid (pseudoplastic), where the fluid viscosity does not have a linear relationship with the rate of shear [11]. Other behaviours of non-Newtonian fluids, such as Bingham or dilatant plastic, also feature in this regard, which have mostly been considered in drilling fluid studies within the literature. A typical polymer flood project involves the addition of polymer fluid to around 1/3 to 1/2 of reservoir pore volume (PV), injected after thorough mixing [10]. Following this, a waterflood is carried out to drive the polymer slug towards the oil bank region of the production wells. This injection is usually maintained over several years until the desired PV is achieved for proper oil recovery. The injected water flood usually flows through the highest permeability layers (least resistance pathway) to the lower pressure zone, which offsets the production well. Fingering usually occurs when oil viscosity inside the reservoir is greater than that of the injected water, which leads to low efficiency in the oil sweep because the injected fluid tends to travel ahead of the oil through the free and porous media of high permeable layers. However, the addition of polymer reduces relative permeability in the reservoir and thereby increases the water viscosity, which in turn helps to increase fractional flow to avoid early breakthrough. Ideally, according to [10,12], a mobility control agent must be resistive to both mechanical and microbial degradation. It should also have a low retention (adsorption) effect on the porous rock and should be insensitive to the actions of O_2_, H_2_S, pH, or oilfield chemicals.

Several developed polymers (synthetic and biopolymers) have been proposed and manufactured over the years, as documented by [12,13]. Some of these have been applied by [14]. However, a review of the literature demonstrates that developed polymer application has been restricted as fit-for-purpose polymeric materials are not readily available, especially in Middle Eastern Asia where harsh reservoir conditions are present. Hydrolyzed polyacrylamide (HPAM) is a widely used mobility control synthetic polymer, but it possesses low viscosity due to weakness in its expansion of polyelectrolyte chains in an ionic solution. HPAM also experiences high sensitivity towards cations, especially divalent ones [15]. In recent times researchers have shifted their focus towards more rigid and stable biopolymers (xanthan, scleroglucan, and schizophyllan) due to their excellent viscosity and good tolerance to harsh reservoir conditions. Amongst the most recently applied forms, scleroglucan and schizophyllan have been proven to possess good resistance to harsh conditions of temperature and salinity but have also been shown to be too expensive for common usage. Contrastingly, xanthan is eco-friendly, economical, and has good qualities when it comes to harsh environments.

Therefore, this paper aims to provide a detailed investigation and comparison of the influence of biopolymers, such as xanthan gum (XG), with synthetic polymers, such as HPAM, based on the above-mentioned chemical and physical properties of these polymers. The first section introduces, in detail, the concept of polyacrylamides and polysaccharides, where HPAM and XG in addition to scleroglucan and schizophyllan are discussed. The second part of the review covers both the physical and chemical properties (characteristics) of specific polymers (HPAM and XG). In this part, rheological properties (shear rate, shear stress, and viscosity) and flow parameters (mobility, permeability reduction, adsorption, and retention) are covered. Furthermore, the screening, design, and optimum concentration criteria for proper selection are highlighted before presenting the key conditions required for application of these polymers. Laboratory, pilot, and field-scale conditions are discussed to support the conditions for these applications. Finally, we present a summary section as a concluding note to the entire review.

## 2. Polyacrylamides and Polysaccharides

### 2.1. Polyacrylamide

**Figure 2:**
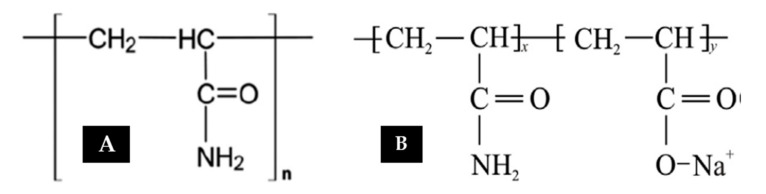
Chemical structure of (**A**) polyacrylamide (PAM) and (**B**) hydrolyzed polyacrylamide (HPAM). Reprinted and modified from [16]. Copyright @Elsevier 2011.

This is a synthetic polymer manufactured based on the polymerization of acrylamide monomers. Polyacrylamide (PAM) is a parent chain used as a thickening agent for aqueous solutions (see Figure 2A). This thickening ability (viscosity) is dependent on the molecular weight ($>1\times 10^{6}\;g/\mathrm{mol})$ and degree of hydrolysis. The degree of hydrolysis is the amide fraction (NH_2_) converted to the carboxylic group (COO-). As reported by [16], the degree of hydrolysis of the amide group to the carboxylic group ranges from 25 to 35%, where PAM serves as the reference model system for modification. Attempts have been made by several authors [17,18,19] to alter chemical structures towards a new acrylamide-based copolymer with improved characteristics of shear resistance, stable temperature, and brine compatibility. In another study, Wever et al. [16] have highlighted how Morgan and McCormick [20] tried to test EOR potential by synthesizing copolymer N,N-dimethyl acrylamide with Na-2-acrylamide-2-methylpropanesulfonate (NNDAM-NaAMPS). They found that for the synthesized copolymer to be stable, it needed to be aged for approximately 1 month at 120 °C. The most commonly/regularly applied copolymer in the oil industry is partially hydrolyzed polyacrylamide (HPAM). This is a copolymer of PAM and polyacrylic acid (PAA) through hydrolysis or copolymerization of sodium acrylate with acrylamide [20]. Figure 2B displays the chemical structure of HPAM, showing straight-chained acrylamide monomers and the occurrence of some hydrolysis. HPAM is a widely used polymer as a result of its good solubility, lower cost compared to alternatives, and its tolerance for mechanical forces within a reservoir during flooding.

### 2.2. Polysaccharide

Generally, biopolymers are either double helix (xanthan) or triple helix (scleroglucan and schizophyllan), depending on the conformation exhibited when present in a solution. This helical structure informs the nature of their rigidity, which impacts their viscosifying power, insensitivity to cations (mono or divalent), and resistance to high salinity and temperature with low mechanical degradation effect, in addition to resistance to degradation due to oxygen and hydrogen sulphide [21,22]. In this work, although primary attention is centered on a comparative study between HPAM and xanthan gum, we, present a brief introduction to other biopolymers (scleroglucan and schizophyllan) to provide the reader with a clearer picture.

#### 2.2.1. Xanthan Gum

**Figure 3:**
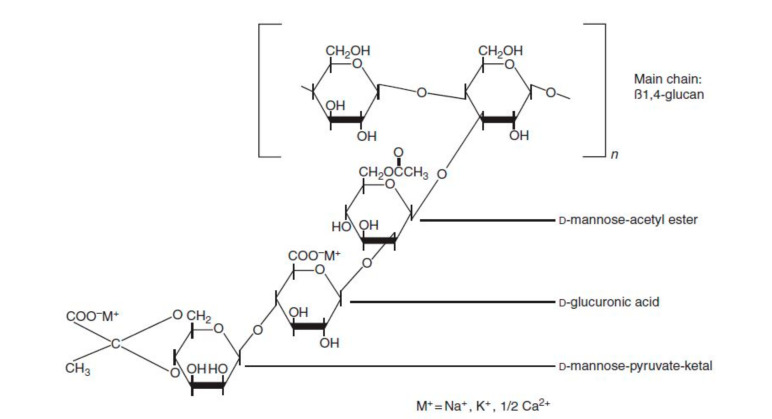
Chemical structure of XG showing both D-Mannose and D-glucuronic acid units linked to the backbone of the glucose, while (M^+^) is the cation binding sites. Reprinted from [24]. Copyright @Elsevier 2012

**Figure 4:**
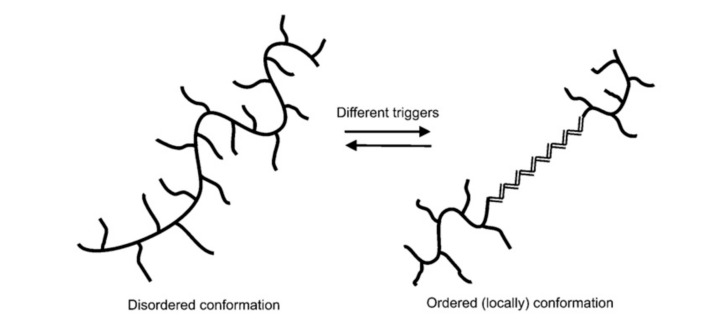
The transition of xanthan gum (XG) polymer. Reprinted from [16]. Copyright @Elsevier 2011.

This is the most common polysaccharide, where it is a green polymer due to the action of its microbes. It is a non-toxic and biodegradable polymer that is commercially produced through the action of Xanthomonas campestris bacterium (a microbial organism) on carbohydrate substrate (glucose or fructose) with a protein supplement and an inorganic nitrogen source. This process is known as fermentation [23]. The chemical structure of xanthan gum is presented in Figure 3, showing a single glucuronic acid unit, two mannose units, and two glucose units of molar ratio 2.0, 2.0, and 2.8, respectively [16,24]. Unlike HPAM, the thickening ability of XG depends solely on its high molecular weight ($2\;\mathrm{to}\;50\times 10^{6}\;g/\mathrm{mol})$ and the rigidness of the polymer chains [25]. Apart from EOR applications, XG has been used in the food and cosmetic industries due to its good gelling effect and non-toxicity [26]. XG operates on a basis that is similar to that of cellulose. The side of the polymer chain comprises of charged moieties (i.e., acetate and pyruvate groups) that make the polymer exhibit polyelectrolytic properties. This property affects the behaviour of XG with the addition of salt because it tends to interact with the ions contained in the solution. Studies have reported [27,28,29,30,31] on the effects of salt addition to XG, finding that when XG chains react with salt (mono or divalent), they undergo a transition from a disordered configuration to an ordered and more chemically stable shape (see Figure 4).

In contrast to HPAM, XG is thermally stable with good resistance to hardness and good salt compatibility. However, it is also biodegradable and has a potential plugging effect when applied in excess [32]. In regards to its biodegradability, a study by [33,34] has shown that salt-tolerant (aerobic and anaerobic) microorganisms can degrade XG, causing it to lose its viscosity strength. Accordingly, the application of formaldehyde serves as an effective biocide to counter this effect but it similarly increases the polymer’s environmental impact [33]. Characterization of XG’s non-Newtonian behaviour (pseudoplastic) can be analyzed theoretically using Herschel–Bulkley (pseudoplastic with yield stress) or Ostwald de Waele (pseudoplastic without yield stress) models [35]. Rheological behaviour (shear thinning) shows that compared to HPAM, XG has a lower power-law index, as will be explained in detail in the course of this work. From an environmental perspective, synthetic polymer usage in EOR is undesirable. Following injection, HPAM either remains in the reservoir or follows oil in water (O/W) dispersion out of the reservoir via a production well, requiring separation for recovery. In optimal cases, most injected HPAM is retained in the reservoir. Due to forthcoming environmental regulations, industrial processes have been mandated to reduce usage and spillage of non-biodegradable polymers, as they strongly contribute to bioaccumulation in the case of unintended environmental release. Therefore, bio-polymer-based EOR formulations have become more appealing.

#### 2.2.2. Scleroglucan

**Figure 5:**
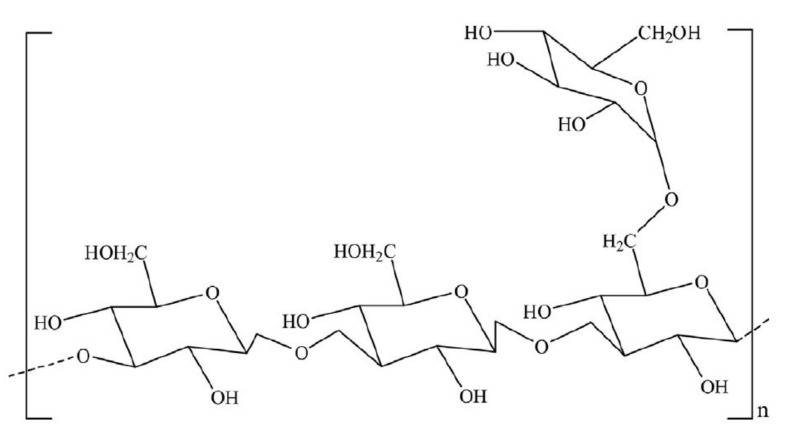
Chemical structure of scleroglucan repeating units. Reprinted from [37]. Copyright @Elsevier 2018.

This type of biopolymer is produced by a fermentation process involving a plant pathogen fungus of the genus *Sclerotium*. Some authors have pointed out that other biopolymers of fungal origin do exist [36] but that potential application in recent times has been based on antitumor effects alluding to the $\left(1,\;3\right)-\beta -D-$glucan family as well as their effectiveness in pharmaceutical industries. Scleroglucan is a rod-like biopolymer with a triple-helical structure. In terms of rigidity, the helical structure exhibited by scleroglucan leads it to behave as a semirigid molecule in an aqueous solution. These repeating structures are linked (linearly) via β−1, 3−D glucose residues, where the side chain is usually attached to every third main chain residue with β−1, 6−D glucose on the backbone (see Figure 5) [15].

The helical structure and the rigidity of the polymeric molecular chain enable it to exhibit superb viscosifying properties, such as good resistance to shear and excellent tolerance to temperature. Furthermore, its non-ionic nature explains how it is highly insensitive to the electrolyte (salts) and how it possesses outstanding durability to pH and mineralogy [37]. This condition has been confirmed in a study by [38], where they investigated the effects of isolation and physicochemical characterization of scleroglucan. They noted that when adjusting the pH range to either highly acidic or moderately basic, the scleroglucan solution remained unchanged. However, they pointed out that a steep decrease in apparent viscosity was observed when pH went above 13. This was ascribed to the decomposition of the helical structure at the highly extreme conditions set for the experiment. In the same study, the authors documented that when NaCl, KCl, CaCl_2_, MgCl_2_, and MnCl_2_ were added to the solution, a slight decrease in viscosity was observed, whereas an increase in viscosity was achieved when adding FeCl_3_. In summary, Fariña et al. [38] have explained that induced interaction within the stranded multi-structure by inorganic salts leads to a drop in viscosity, whereas breakage of glycosidic linkages due to FeCl_3_ presence from aggregation and gelation might cause increases in viscosity.

In addition to its high stability, scleroglucan behaves like a good viscosifying agent at low concentrations because of its chain rigidity and high molecular weight [39]. Rivenq et al. [15] have explained that application of scleroglucan as a mobility control agent could be employed where XG experiences a transition from a rod-like confirmation to a random coil because of its extreme uniqueness in high temperature and salinity. Akstinat [40] reported scleroglucan to remain stable at 80 °C in a highly saline environment. Davison and Mentzer [41] have investigated the retention and mobility reduction of over 140 polymers via a porous medium, determining that scleroglucan at a high temperature (90 °C) provides the best potential. Another study by [42] notes that both with or without additives, scleroglucan exhibits better thermal stability than XG over 700 days. Contrastingly, Ryles [43] has provided a contrary opinion of scleroglucan, observing a degradation of scleroglucan within 3 months of application at 90 °C. The major disadvantage of scleroglucan as reported by [44] is its plugging and filterability tendency. However, some researchers have been able to solve this problem through biosynthesis modification and post-treatment [45]. Based on its rheological study, scleroglucan behaves as pseudoplastic (shear thinning) with an exponential order between the polymer concentration and apparent viscosity [37].

#### 2.2.3. Schizophyllan

**Figure 6:**
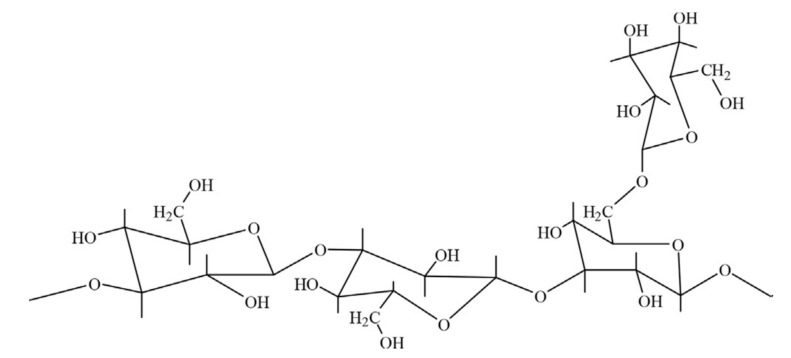
Chemical structure of an schizophyllan. Reprinted from [37]. Copyright @Elsevier 2018.

Schizophyllan (SPG) has been identified as a neutral, uncharged, extra-cellular polysaccharide that is produced from the fungus *Schizophyllum commune.* Just like Scleroglucan, it has received significant attention for application in polymer science (oil industry), biomedical studies (cancer treatment), and food preservation because of its outstanding molecular conformation, non-toxicity, eco-friendliness, and antitumor potential [46,47]. SPG’s repeating chemical structure (see Figure 6) is comprised of linearly linked $\beta -\left(1-3\right)-D$ glucose residues with one $\beta -\left(1-6\right)-D$ glucose side group attached to every three main chain residues [48]. In terms of identity, both scleroglucan and SPG are similar but are produced from different fungi. Fang et al. [49] have documented that SPG, scleroglucan, and lentinan all belong to the family of $\left(1-3\right)-\beta -D$ glucans with branched $\left(1-6\right)$ glucose. However, there are chemical differences between SPG and the scleroglucan family in their degree of branching [49]. SPG’s rod-like cylindrical helical structure and its stiff confirmation provide it with excellent rigidity and stability, which renders it a good viscosifying agent for enhanced oil recovery [50].

Schizophyllan has an excellent ability to withstand remote conditions such as high temperature and salinity, which can be attributed to its intermolecular interactions and stable helical conformation via hydrogen bonds [37]. Furthermore, the free charge exhibited by its backbone leads to low absorption of SPG on rock surfaces [51]. It has been reported that less absorption of SPG will be recorded as temperature decreases and as salinity increases [39,51]. In contrast to scleroglucan, which has been theoretically analyzed using an exponential model, the rheological properties of schizophyllan have been shown to exhibit the same behaviour as xanthan gum (pseudoplastic with shear-thinning characterized using a power-law model) [48]. According to existing studies [52,53], rheological data indicate that biopolymer concentration is vital, wherein low concentration, the solution of SPG behaves as a viscoelastic material, while at high concentration, it maintains a solid-like state.

## 3. Physical and Chemical Characteristics of Green Polymers over HPAM

In this part of the study, detailed characteristics of XG will be compared to those of HPAM solutions. This will be done by initially comparing the rheological parameters of both polymers. Following this, graphs for retention, inaccessible pore volume (PV), and permeability reduction of both types of polymers will be compared. Then, the effect of both polymers on mobility control is presented and the behaviour of both polymers regarding temperature and salinity will be discussed. Finally, a brief discussion on viscous fingering is highlighted before proceeding to the economic aspects, criteria, and conditions for applying these polymers at lab-scale, pilot-scale, and field-scale.

### 3.1. Rheological Properties: Shear Rate, Shear Stress, and Viscosity

**Figure 7:**
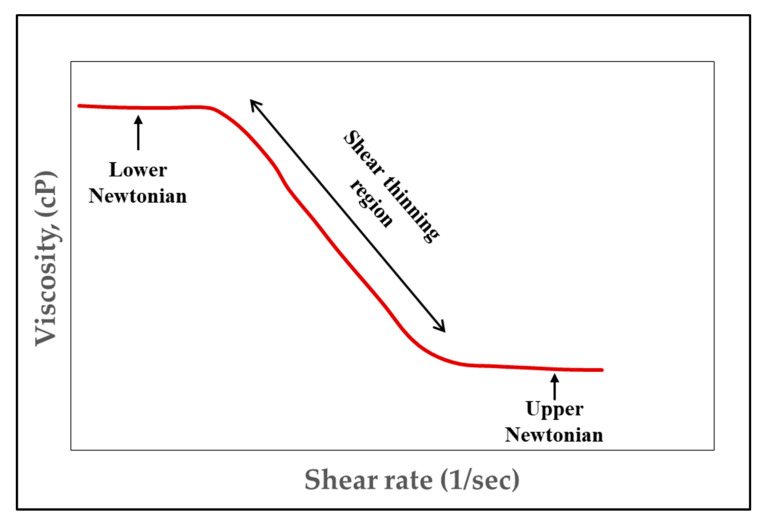
Rheology of a shear-thinning fluid (Log-Log plot). Reprinted and modified from [2].

Fluids can be either divided into Newtonian or non-Newtonian fluids. A Newtonian fluid has a linear relationship between shear rate and shear stress, whereas non-Newtonian fluids have a nonlinear relationship between shear rate and shear stress. HPAM and XG polymers used in EOR applications are non-Newtonian fluids [11]. Polymers used in EOR usually have shear-thinning rheological properties. In rheology study, shear-thinning is sometimes considered synonymous to pseudoplastic behaviour of fluid because it is a property which describes fluid behavior when subjected to applied stress. This means that the apparent (when considering different composition) viscosity of the polymer solution decreases when the shear rate is increased, as shown in Figure 7. The reason for this occurrence is due to polymer molecules arranging themselves within the shear rate field where internal friction is reduced [2]. In addition to this, at a low shear rate value, the polymer solution in Figure 7 exhibits a homogeneous sequence (lower Newtonian), whereas when shear increases, the polymer’s apparent viscosity decreases, giving rise to a power law. This region (shear thinning), which obeys the power-law index, is expressed using the Ostwald de Waele model, as shown in Equation (3). A continuous increase of shear rate makes the polymer solution, again, behave as a Newtonian fluid (upper Newtonian).

(1) $$\tau =\mu \times \frac{du}{dy}$$

(2) τ=μ×γ

(3) $$\tau =K{\left(\gamma \right)}^{n}$$

where τ, γ, μ, K, and n, represent shear stress, shear rate, dynamic viscosity, consistency index, and power-law index, respectively.

**Figure 8:**
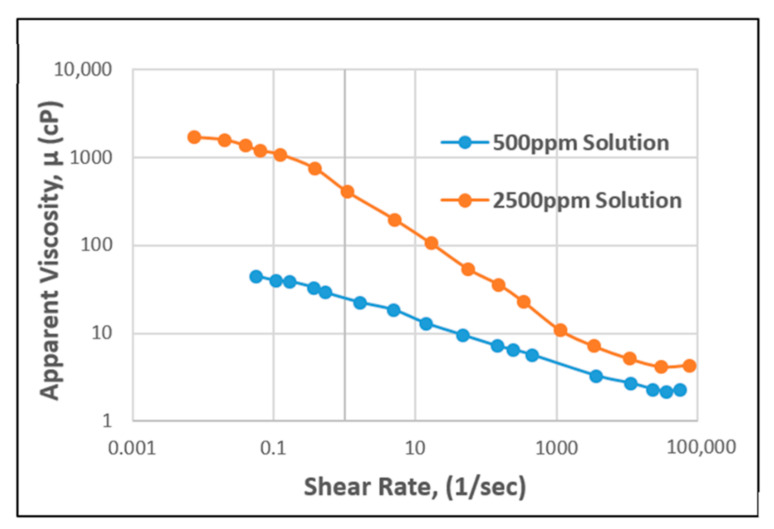
Apparent viscosity vs. shear rate of HPAM. Reprinted and modified from [54].

**Figure 9:**
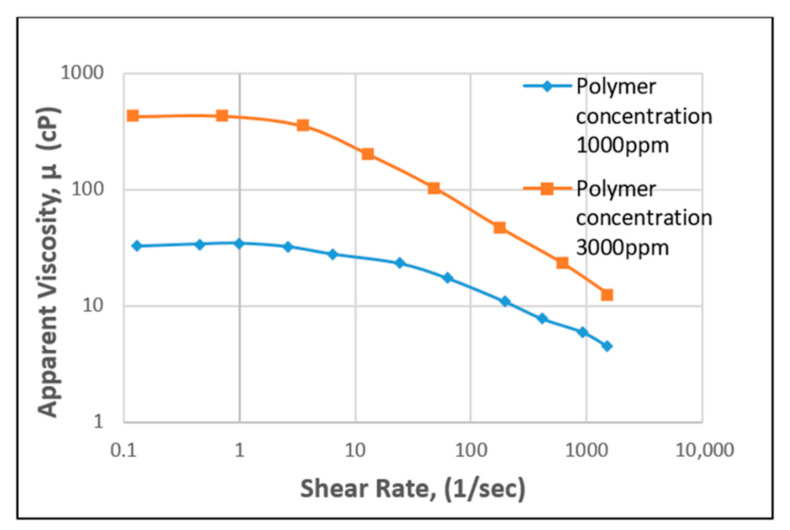
Apparent viscosity vs. shear rate for XG. Reprinted and modified from [55].

A plot of apparent viscosity against shear rate for HPAM polymer behavior is presented in Figure 8, with differing concentrations. From the graph, it can be observed that as the shear rate for the polymer increases, the apparent viscosity decreases, as based on experimental lab data [54]. At higher HPAM concentrations, a higher viscosity can be observed, indicating the good performance of the polymer during flooding. Consequently, the same plot was made for XG (see Figure 9), showing that high concentration of this biopolymer achieves high viscosity compared to low concentration. However, it is worth noting here that with the same experimental conditions and settings, XG behaviour will require a lower shear rate (polymer extension) than the HPAM to achieve the same apparent viscosity.

**Figure 10:**
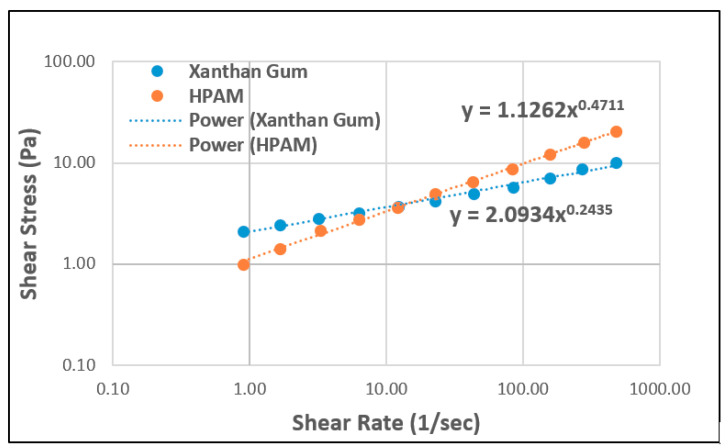
The shear rate vs. shear stress of HPAM and XG. Reprinted and modified from [57].

Figure 10 shows a case of a combination of shear stresses of both polymers as a function of shear rate at 2.1 wt.% total dissolved solids (TDS) brine and 25 °C. The shear-thinning behaviour of both polymers can be observed. The results show a power-law index of 0.47 for HPAM, whereas the power-law index for XG is roughly 0.24. This indicates stronger shear-thinning because the conformational status exhibited by polysaccharide molecules makes them more rigid [56].

#### 3.1.1. Effect of Shear Rate

**Figure 11:**
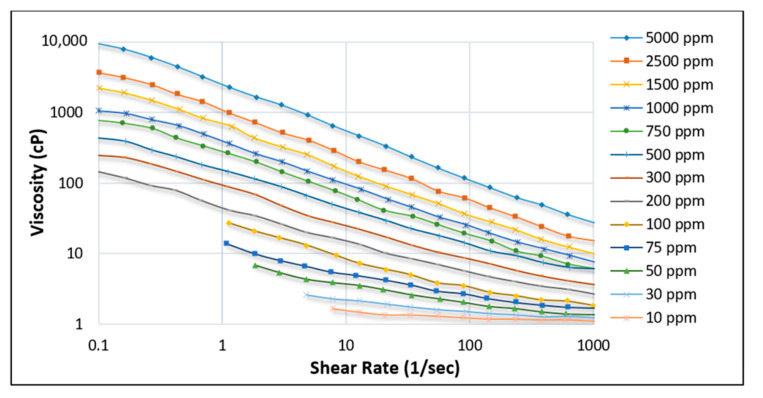
Effect of shear rate on the viscosity of HPAM solution at various concentrations [58].

Kamal et al. [11] and Scott et al. [32] have provided brief overviews of the rheological properties of HPAM across various parameters, including changes in polymer concentration, temperature, and salt. Changes in polymer concentration indicate that with a higher concentration of HPAM polymer, the effect of shear rate becomes more evident (see Figure 11) on the decline in viscosity of the solution, as observed with the case of 5000 ppm concentration.

**Figure 12:**
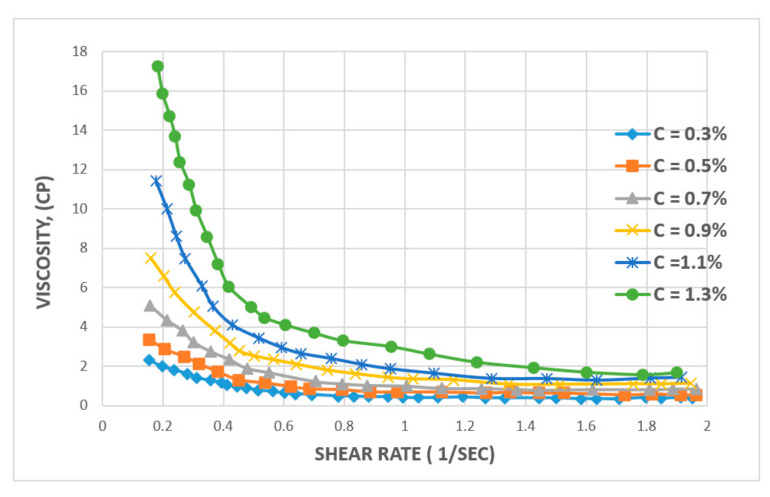
Effect of shear rate on the viscosity of XG solution at various concentrations. Reprinted and modified from [35].

Similarly, when the effect of concentration is analyzed for the XG biopolymer, Ghoumrassi-Barr and Aliouche [35] have reported that increasing the concentration of xanthan leads to a slight initial increase in shear rate and a drastic impact on the viscosity of solution (See Figure 12). However, after increasing the shear rate to 0.8 sec^−1^, the viscosity of the solution stabilizes and remains as high as 2.0 cP. Contrastingly, in the case of HPAM, its viscosity continues to decrease steadily and steeply with increasing shear rate, where xanthan shows better stability against shear rate for polymer viscosity versus shear rate behaviour.

#### 3.1.2. Effect of Salinity and Temperature

Salinity and temperature play a vital role in understanding the rheological behavior of polymer solutions at various concentrations. However, effects are specific to polymer type, especially when it comes to HPAM. When PAM hydrolysis occurs, a negative charge on the backbone of the polymer chain that is responsible for prominent stretching (due to electric repulsion) affects the ionic forces in the salt [59]. At low salinity, a negative charge from divalent ions (Ca^2+^) initiates repulsion, causing the polymer chain to stretch. Individually stretched polymer molecules occupy more space, leading to an increase in the apparent viscosity of the polymer. However, one study [60] has indicated that the degree of hydrolysis must not exceed a certain threshold due to sensitivity to salinity and hardness of the brine. To reduce this effect, a monovalent ion (NaCl) can be added to the polymer solution at higher ionic strength. This creates a barrier on the double layer of the electrolytes where the extension reduces intermittently.

**Figure 13:**
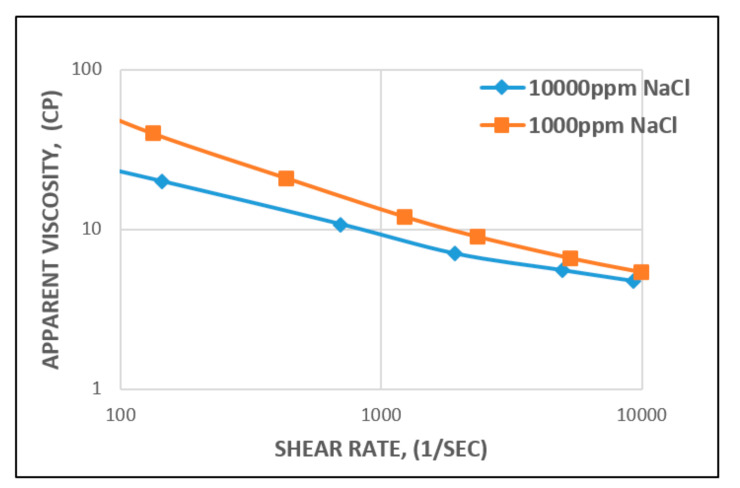
Influence of salinity (Na^+^) on the viscosity of HPAM at different concentrations.

This means that as the concentration (high salinity) of the monovalent (Na^+^) electrolyte increases, the extension of the polymer chains decreases (Figure 13), leading to a decrease in viscosity [61].

**Figure 14:**
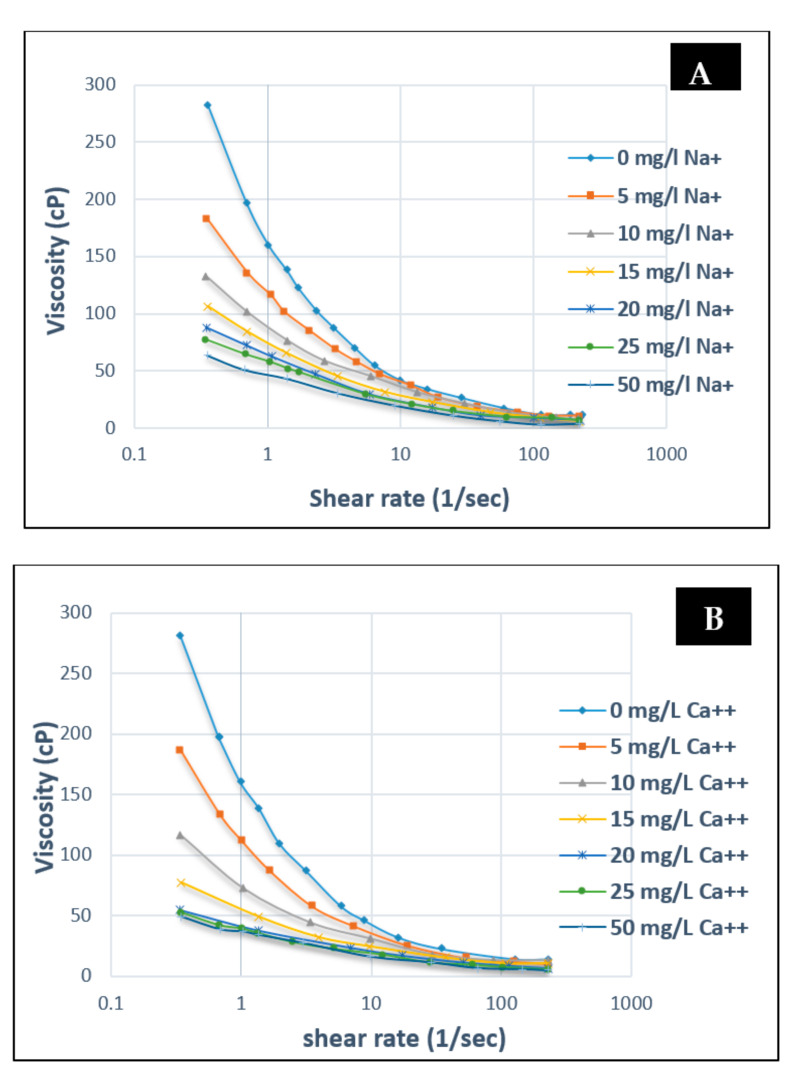
Influence of salinity (**A**) Na^+^ on the viscosity of XG and (**B**) Ca^2+^ on the viscosity of XG at different concentrations. Reprinted and modified from [63].

Contrary to HPAM, electrolyte addition (mono or divalent) to XG will render the polymer chain as more stable and rigid [56,62]. Zhong et al. [63] have studied the rheological behaviour of XG solution (600 mg/L) at various concentrations using Na^+^ and Ca^2+^ ions. They found that the initial addition of cations to a free XG solution had a significant effect on viscosity decrease, whereas further addition had no impact on the viscosity. Figure 14A shows that when 0 mg/L to 25 mg/L of monovalent ions are added at a 0.5 s^−1^ shear rate, a 72% (282 to 80 cP) decrease in viscosity was recorded. However, an additional 25 to 50 mg/L only provided a 5.7% decrease (80 to 64 cP). The same result was found in the case of the divalent ion (Figure 14B). This similarity indicates that the impact of electrolyte (mono or divalent) addition at various concentrations for XG is insignificant.

**Figure 15:**
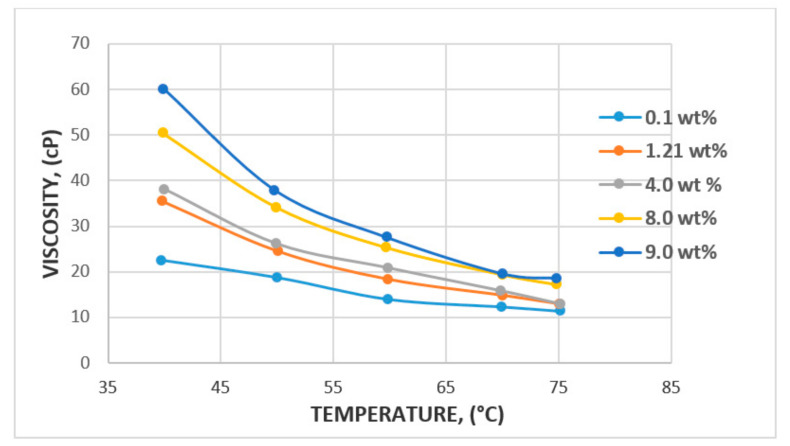
Effect of temperature on HPAM. Reprinted and modified from [6].

**Figure 16:**
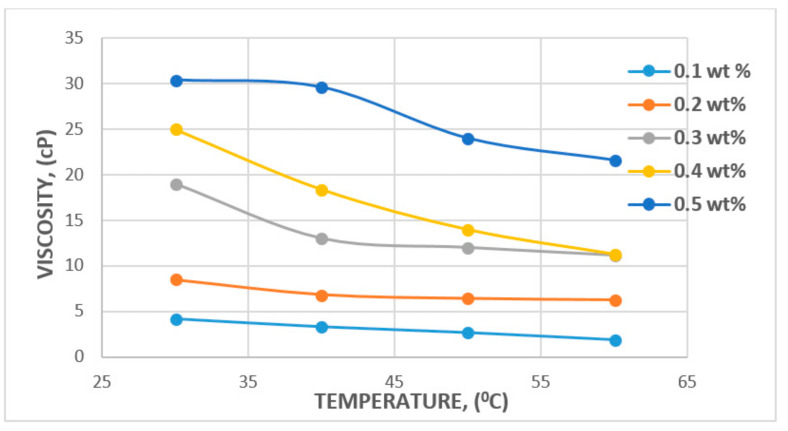
Effect of temperature on XG. Reprinted and modified from [26].

In the case of temperature, some studies have reported 100 °C to be the reservoir temperature that a polymer flood can withstand [64], with a median temperature of 46.1 °C reported by [65]. A major factor that could affect temperature change is the degradation potential of the polymer backbone or potential hydrolysis [66,67]. The major challenge faced in relation to degradation is the molecular weight of polymer and viscosity modification. However, a study by Muller [66] has found that PAM hydrolysis can occur at as low as 60 °C and that conditions such as hardness and oxygen contamination might contribute to the degree of hydrolysis at any given temperature. Following these findings, Kierulf and Sutherland [68] tested commercial XG and found that the XG solution remained constant for about 2 years at 80 °C. It was later observed that it was not until the temperature got to 100 °C and above that solution viscosity began to decrease. This, in addition to other studies by [11,69], has led to the conclusion that although both polymers are affected by temperature, the XG polysaccharide is more resistant to temperature and salinity compared to the HPAM polymer. The effect of temperature and viscosity on HPAM and XG polymers is shown in Figure 15 and Figure 16, respectively. Figure 15 represents HPAM, showing an instant steep decrease in viscosity with temperature as concentration increases, whereas, in Figure 16, a gentle decrease in XG is recorded with increase in temperature and concentration at the initial stage before a steep decrease at the later stage of the flooding process. Hence, HPAM reacts to temperature effects faster as compared to XG. This, therefore, makes XG a better candidate for a high temperature environment. A summary of some reported literature with the above discussed properties is presented in Table 1.

### 3.2. Polymer Flow Properties

#### 3.2.1. Permeability Reduction and Polymer Mobility

The essence of a polymer flood is to improve sweep efficiency by increasing the mobility of an injected fluid. The concentration of the injected fluid may be increased depending on reservoir conditions. However, increasing the concentration of a polymer may not be advisable in many cases because of the high cost involved with the high concentration of a polymer, as well as injectivity issues (high pressure) associated with injecting highly concentrated polymers into a wellbore [65]. Instead of doing this, Delshad et al. [7] have suggested using a polymer with a high molecular weight, suggesting it to be more economical and highly effective to use a high molecular weight polymer at a lower concentration rather than a polymer with higher concentration to achieve a high viscosity rate that will support the sweep efficiency of the polymer. However, this method is not without reservation because a high molecular weight polymer could increase inaccessible pore volume (IPV), which is the pore space that is not accessed by the injected polymer. The International Union of Pure and Applied Chemistry (IUPAC) have classified reservoir pores into micropores (<2 nm), mesopores (2–50 nm), and macropores (>50 nm) [79]. Therefore, increasing the molecular weight of a polymer may prevent the polymer molecule from flowing through the pores of reservoir rock due to size difference. Green and Willhite, [2] have illustrated that depending on polymer type and porous medium, XG biopolymer IPV ranges between (20% to 31%), whereas HPAM IPV ranges between (0.18% to 0.24%). This signifies that HPAM is more suitable than XG because the small void space in the porous rock will not be filled by the polymer solution.

Permeability reduction is caused by polymer retention, which reduces the apparent permeability of the rock, depending on polymer types, amount of retained polymer, and finally, the average size of polymer relative to reservoir rock pores [80]. Reduced permeability can be obtained after using a polymer solution to displace a porous medium, followed with a brine solution, before measuring the permeability to brine after displacing all mobile polymers from the rock. In general, the mobility (λ) of any fluid is defined as the ratio of effective permeability of fluid to its viscosity. Al Quraishi and Alsewailem [13] have identified two basic factors used to characterize polymer mobility theory: resistance factor (RF) and a residual resistance factor (RRF).

The RF (mobility ratio) is the ratio of the displacing fluid (brine mobility) to that of the displaced fluid (polymer solution) under the same flow conditions (see Equation (4)). For instance, RF = 5 denotes that it will be five times more difficult to flow a polymer solution through a system, or that five times the amount of pressure is required to inject the polymer in comparison to water.

(4) $$RF=\frac{\lambda_{w}}{\lambda p}=\frac{\left(\frac{k}{\mu }\right)waterflooding}{\left(\frac{k}{\mu }\right)polymerflooding}=\frac{\Delta P_{polymer}}{\Delta P_{water}}$$

where $\lambda_{w}$ and λp represent mobility ratios for water and polymer, respectively, k and μ denote permeability and viscosity for water and polymer flood, respectively, and where ΔP is the respective pressure drop. The numerator describes the mobility of the brine as the denominator describes the mobility of the polymer compared to oil mobility, which is commonly used in water flooding. RRF describes the ratio of rock resistance to the flow of the initial water injected behind the polymer solution (see Equation (5)). For instance, an RRF value of five denotes that roughly 25% of the initial permeability of the rock can be obtained after a polymer flood. This percentage, therefore, shows the amount of reduced permeability in the rock due to adsorbed (retained) polymer molecules.

(5) $$RRF=\frac{\left(\frac{k}{\mu }\right)before\;polymer\;flood}{\left(\frac{k}{\mu }\right)\;after\;polymer\;flood}=\;\frac{\Delta P_{water\;after\;polymer\;flood}}{\Delta P_{water\;before\;polymer\;flood}}$$

**Figure 17:**
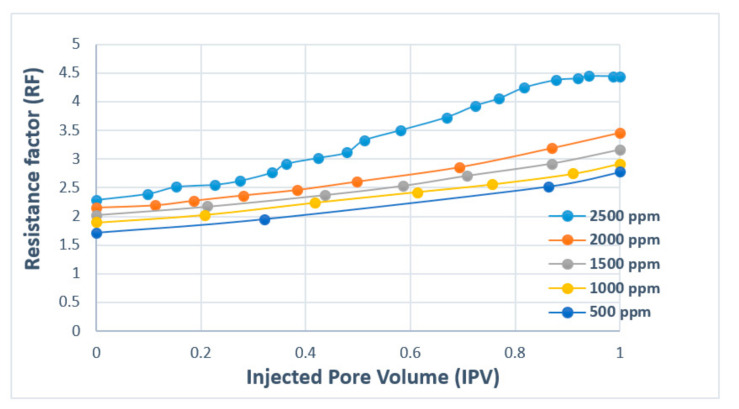
Resistance factor (RF) versus injected pore volume (PV) for HPAM. Reprinted and modified from [72].

**Figure 18:**
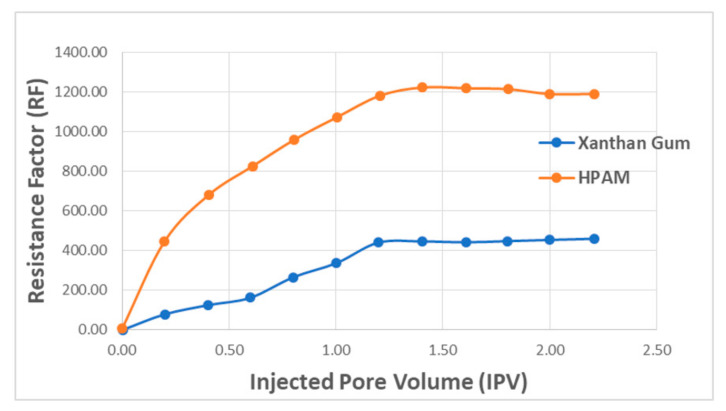
RF versus injected PV for both the polymers. Reprinted and modified from [57].

Figure 17 presents polymer concentration as a function of RF and injected pore volume. The results show that with increasing injected pore volume, RF value also increases with a higher concentration of polymer. This means that greater adsorption (reduction in permeability) will occur as the injection of the polymer continues. The comparison plot for HPAM and XG is presented in Figure 18. The results denote that RF increases with injected fluid volume until a plateau is reached, where stabilized RF values can be achieved after injecting 1.2 PV of the polymer solution. For better performance in polymer flooding operation, it is desirable to have a higher RF as it prolongs the characteristics of the polymer flood. However, a higher residual factor is associated with a polymer’s tendency to adsorb, thus partially blocking porous media. Therefore, a tradeoff between adsorption and prolonged flood operation is required in order to obtain an optimal polymer flooding operation.

#### 3.2.2. Retention and Adsorption of Polymer

Polymer retention entails interactions between polymer molecules and a porous medium, leading to the entrapment of polymers on the rock surface [81]. This occurrence usually causes flow impediment (reduction in permeability) as some of the injected polymers are lost to the rock surface, which could lead to rock damage (plugging) and affect the ease of sweep efficiency and oil recovery. Polymer concentration from a flowing polymer solution could be (1) adsorbed on the surface of the rock [82]; (2) mechanically trapped by the narrow pore throats relative to the molecule size [83]; (3) hydrodynamically retained as caused by drag forces trapping the polymer molecules in stagnant flow zones [58]. As polymer adsorption takes place via physical means, Ferreira and Moreno [58] have noted that this should be regarded as a reversible process in principle. However, most researchers theorize that polymer adsorption is an instantaneous and irreversible process [84].

**Figure 19:**
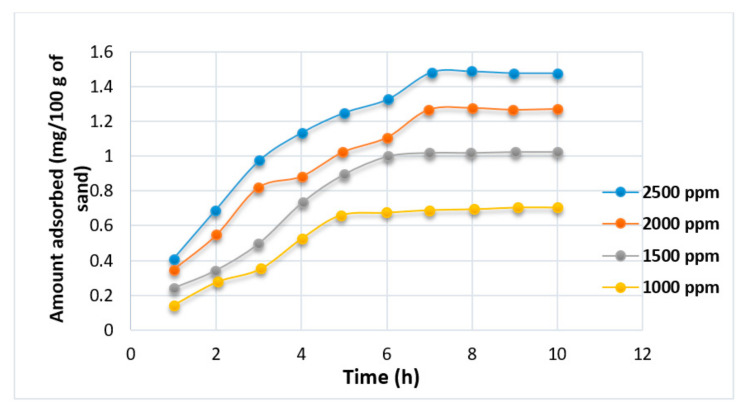
Effect of adsorption on HPAM. Reprinted and modified from [72].

Studies by [82,83,85,86,87,88] have shown that polymer retention in a porous medium largely depends on the concentration of polymer, temperature, flow rate, size of polymer slug, brine salinity, absolute permeability of the rock, oil saturation, mineralogy, and rock wettability. The effects of HPAM polymer concentration on adsorption (see Figure 19) have been demonstrated by Mishra et al. [72], noting that higher concentrations (1000 to 2500 ppm) increase polymer adsorption significantly until a point where adsorption became saturated (constant) with time. This shows that polymer adsorption possesses a linear relationship to polymer concentration. Unfortunately, the study by Mishra et al. did not detail the effect of using an XG biopolymer on the surface of porous media. Theoretically, it is expected that the adsorption should be lower than that of HPAM because, as highlighted by [2], biopolymer retention is generally between (38 to 78 Ibm/acre-ft), whereas for HPAM it is between (35 to 1000 Ibm/acre-ft). This confirms that less adsorption will occur when XG is employed.

#### 3.2.3. Viscous Fingering and Its Effect

Viscous fingering is defined as the systematic formation of patterns due to instability between two fluids in a porous media. It usually occurs when a thin linear channel of fluid pushes a more viscous fluid, leading to a formation of a finger-like pattern of channels [89]. This phenomenon, dated back to the late 1950s [90], has received significant attention because the limiting factor is the recovery of oil. In EOR, viscous fingering is mostly associated with miscible gas injection processes because of the premature breakthrough and high mobility associated with CO_2_ gas. Green and Willhite [2] highlighted that the relatively low density and viscosity of CO_2_ as compared to reservoir oil are the major determinants of viscous fingering. However, water alternating gas (WAG) and foaming solutions have been employed to combat this challenge as regards the volumetric sweep efficiency control and the reduction in CO_2_ mobility, respectively [91,92,93]. In the case of polymer flood, temperature, concentration, and viscosity are the major factors associated with viscous fingering. The HPAM polymer will be affected more because of its lower resistance to a higher temperature as compared to XG. Subsequently, it is a fact that an increase in temperature will cause a decrease in viscosity, which will further affect the mobility of the injection fluid, hence leading to fingering in the reservoir. However, since the optimum polymer concentration is highly dependent on the shear strength, salinity, temperature, and adsorption power on the rock surface [94], the XG biopolymer, which is an excellent polymer when compared with HPAM based on these properties, will resist the effect of viscous fingering more in the reservoirs.

### 3.3. Cost (Per Kilogram)

For any given application, the best choice of polymer not only depends on performance but also on economic value. In this regard, it is worthy to mention that polymer cost plays a vital role in field operations, notably in oil fields where a large amount of polymers is required for flooding. Secondly, uncertainty and change in polymer costs can impact field operation, which, if not addressed, could amount to the shutting down of a polymer plant. In Chang’s [95] article, entitled “Polymer flooding technology-Yesterday, Today and Tomorrow”, the author highlighted seven key factors affecting overall polymer flooding economics. These factors include (1) crude oil price; (2) capital investment cost (CAPEX); (3) chemical cost; (4) operating cost (OPEX); (5) process effectiveness; (6) taxation; (7) environmental control.

Taxation is dependent on the region where the field is situated as each country has a different policy for tax, whereas process-dependent factors, such as the cost of the chemical, depending on the success of the screening and design criteria (Section 4) for a polymer flood. For instance, while XG’s chemical treatment costs are higher than HPAM, XG is more stable in the global market as HPAM is derived from petroleum products whose costs are sensitive to crude oil prices. However, there is an additional treatment cost (biocide application) associated with XG due to its biodegradability, which is also significant but depends on the location, well spacing and depth, and the age of the field [69].

The cost of both polymers has decreased since the early 1980–1990s when the chemical EOR process was booming. According to Llano et al. [96], the cost of an HPAM product ranges between approximately USD 5–6/kg. Comparatively, as reported by Aspinall [97], the cost of XG was approximately USD 5–11/kg in the 1980s. However, current market prices suggest that an XG biopolymer can cost around USD 2–3/kg [69]. This shows that the market is shifting towards biopolymers as they are more environmentally friendly, more efficient, and effective at surviving in much higher salinity.

## 4. Screening, Design Criteria and Optimum Polymer Concentration

For any polymeric materials to be considered for CEOR polymer flooding, useful information about the nature of the reservoir, flooding mechanisms, and other targeted flooding processes must be well understood. Various polymer flooding applications have been reported, where it would be unscientific to inject polymer materials or carry out scaled production within an oil well before characterization of the relevant properties is completed.

### 4.1. Screening Criteria

General knowledge for polymer screening (see Table 2) has been benchmarked based on classifications as reported by [2,98]. However, other additional criteria have been reported, as this process keeps evolving. Some of these have been captured in detail by [65,69].

The most common parameters to be considered in this regard are the type of crude oil (low and medium), formation type, and temperature for biopolymer flooding. Other criteria have been cited during the discussion in previous sections.

### 4.2. Design Criteria

**Figure 20:**
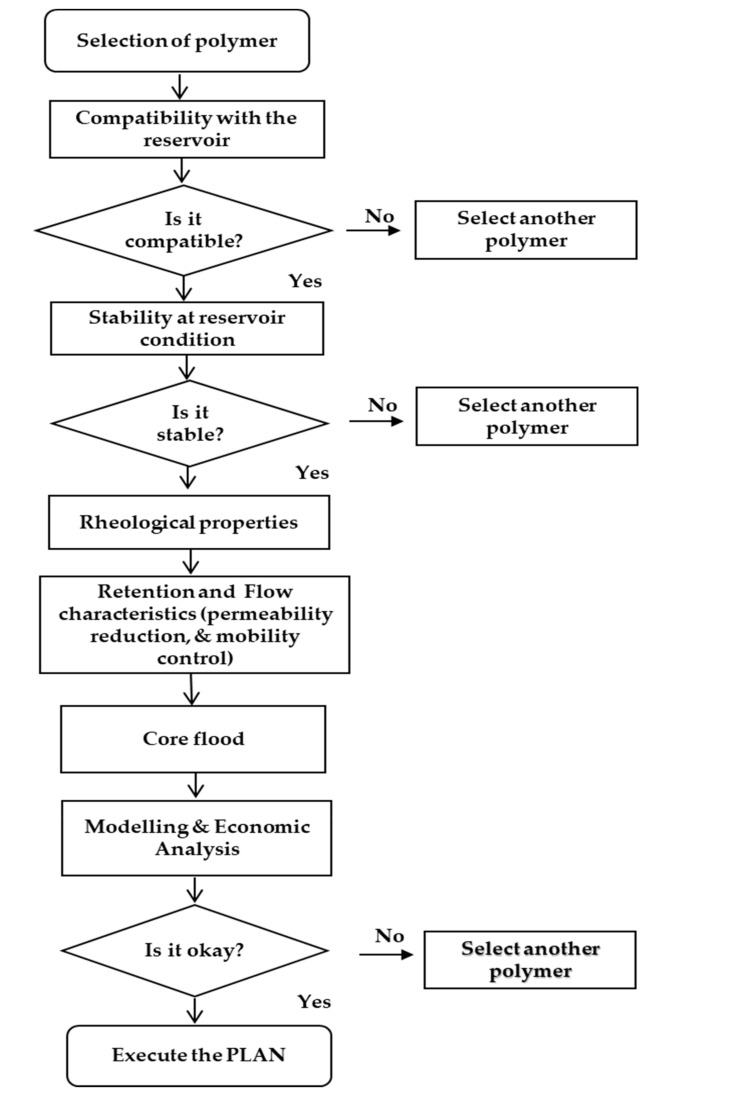
Schematic for polymer design criteria.

The first step in polymer design criteria is to decide whether or not the field is good enough to be suitable for polymer flooding. Further, critical questions (for all cases; lab-scale, pilot-scale, and field-scale) as described in Figure 20 must be considered. These and others should be addressed before proceeding to the design draft. Suitable reservoirs (mostly sandstone) for polymer flooding usually have the following characteristics. First, they have poor volumetric sweep efficiency of water flooding and a low recovery from water flooding compared with similar ones. More so, when water flooding is applied, their breakthrough is reached very quickly [2]. In addition to this, a high water oil ratio (WOR) exists throughout the life of the waterflood system. Determining the mobility ratio of the water flooding also plays a vital role because it helps to control and estimate the mobility problem at the microscopic level.

An increase in mobility ratio (M > 1) leads to the fingering effect as the microscopic and volumetric sweep efficiency is affected (decreases). However, when the mobility ratio is (M < 1) the fractional fluid flow behaves like a piston, thereby decreasing water mobility and resulting in a high sweep efficiency of the oil. Additionally, through various means of distinguishing reservoir heterogeneity (such as the Dykstra–Parsons coefficient and so on), information about the reservoir through core analysis and displacement calculations can determine sweep efficiency and whether polymer flood should be applied [98]. Lastly, a mathematical model can be used to predict the outcome or performance level as the degree of complexity for polymer flood varies across reservoirs.

### 4.3. Determination of Optimum Polymer Concentration

The optimum polymer concentration selection according to [94] should be considered as the main step in designing a polymer flooding project since it affects both the technical and economic feasibility of the project. In the case of a surfactant where the critical micelle concentration (CMC) region and the hydrophilic lipophilic balance (HLB) informs the optimum surfactant concentration selection [99], several factors, such as shearing, temperature, salinity, and adsorption, influence the optimum polymer concentration and viscosity required for achieving favourable mobility ratio [2,94]. Thus, the impact of these factors should be considered while selecting the optimum concentration. Moussa [94] highlighted that a polymer’s viscosity changes when exposed to any external force or stress and it is affected by temperature, the magnitude of the force, and the nature of the solution itself, investigating the relation between shear rates and polymer viscosity. Additionally, the effect of temperature could accelerate oxygen free radical reactions which could affect the polymer performance at 23, 49, and 60 °C [100]. In the case of rheology, Wang and Caudle [101] discussed that concentrated polymer slug is vital for efficient oil recovery. However, the authors highlighted that increasing the polymer concentration, in contrast, could affect the required slug. According to Sarem [102], when a polymer solution flows through porous media, its large molecules will adhere to the rock surface as they will not be able to pass through narrow pores. This behaviour is desired to a certain extent as when polymer molecules attach to the surface, they stretch out and plug the path of water, thus its mobility is lowered. However, it is not favourable for the polymer to adsorb permanently or slowly as this may result in excessive loss of the polymer or small flow resistance, which will affect the profitability of the polymer flooding project. Omar [103] investigated the effect of adsorption on polymer losses and concluded that when polymer molecules adsorb on the rock surface, the concentration of the solution leaving the pores is lower than the concentration of the initial polymer solution injected. This reduction in polymer concentration can be used as a measure of the adsorption. Thus, polymer adsorption increases the polymer resistance to flow and loss of polymer. In a nutshell, both XG and HPAM undergo the same behaviour when it comes to optimum concentration because all the above factors are imminent to the characteristics of every polymer. However, the degree to which both polymers react to some of these characteristics is different, as earlier discussed.

## 5. Conditions for Polymer Applications

Different measures must be implemented to ascertain if a suggested EOR technique is suitable for a candidate reservoir based on the secondary recovery stage. Notwithstanding this, before some of these techniques (polymer flooding in this case) can be applied to field cases, experimental or laboratory applications and pilot testing should be conducted to ascertain the levels of success and difficulty that may be faced. Therefore, this section of the paper outlines the various conditions that should be met in regard to both HPAM and XG before field application is carried out.

### 5.1. Lab-Scale Application

In a recent review by Saleh et al. [64], the authors found that the highest amount of collected data on polymer flooding was based on bi-annual EOR survey publications by the Oil and Gas Journal. Though information about both completed and ongoing processes have been covered, vital factors, such as the formation of water salinity, type, and polymer concentration, have not been fully captured. In addition to this, the field data do not account for recent technological developments relating to polymer flood. To this effect, Saleh et al. [64] captured a total of 329 polymer flood projects specifically using the experimental analysis to determine the best acceptable conditions for polymer flooding to be employed. They found roughly 61% of HPAM, 28% of XG, and 11% of other associated polymers to have been tested for laboratory application with porous medium, ranging from sandstone cores (53%), carbonate cores (8%), sand-packs (30%), and micromodels (9%). Table 3 displays a typical range of laboratory conditions for polymer applications.

With regards to Table 3**,** it appears that most published works on experimental polymer flood have used higher HPAM concentrations than XG and other polymer categories. This could be ascribed to the fact that at the same salinity level, XG and OA will serve as better viscosifying agents at a lower concentration than HPAM [91]. Consequently, XG is more resistant to mechanical degradation [104] and can withstand high salinity and high temperatures [75]. Another contrary opinion has been reported as to the application of XG and HPAM based on oil viscosities. A study by [75,105] on a laboratory scale found that XG could displace heavy oil with >450 cP of viscosity. This shows that the study by [64] presented in Table 3 might have too greatly limited viscosity range for XG application.

### 5.2. Pilot-Scale Application

Under the case of lab-scale application, Saleh et al. [64] jointly analyzed over 70 pilot polymer flood studies, covering countries such as Austria, Oman, India, Argentina, Germany, Brazil, Canada, and the USA. The result of the suggested pilot conditions is summarized and presented in Table 4. No clear evidence between the HPAM and XG polymers was recorded, suggesting that either polymer could be employed when testing the recovery potential for a pilot scale. However, it is worth noting that most reported pilot-scale studies used a higher molecular weight (MW) and a relatively low concentration of polymers when compared to Table 3. This claim, according to Choi et al. [106], may be associated with project economics. In the case of brine salinity, the pilot-scale application is usually based on a low average value of 500 to 120,000 ppm, which is far less compared to lab-scale (250 to 350,000 ppm). Torrealba and Hoteit, [85] also suggest that most designed pilot tests achieve about 0.15 PV per year during implementation.

### 5.3. Field-Scale Application

The sweep efficiency and oil recovery offered by polymer flooding position this technique as the most common one applied for mobility control. Amongst other countries, China has successfully benefitted from this process. The Daqing oil field in China yielded an additional 22.3% of oil OOIP with a sequential decrease in water production during 12 years of polymer flooding [107]. Many more applications in several fields (Marmul, Bohai Bay, Viraj, Sanand, Pelican Lake, Albrecht, El Corobo) in countries such as Oman, China, India, USA, and Argentina have similarly been reported [60,65,70,76,83,95,96,107,108,109]. These results further show that regardless of the type of polymeric material employed, success has been achieved when conducting polymer flooding [109]. However, this field of application is largely based on the condition of the reservoir and many unforeseen circumstances could arise during production. To this effect, we have presented summarized data for field conditions in polymer application, specifically related to XG and HPAM (Table 5), based on the study by [64,65] with recent modifications from [32]. In addition to this, a summary of some key parameters for field cases where XG and HPAM have been implemented especially in sandstone reservoir is presented in Table 6.

### 5.4. Oil Fields in the Middle East

Several literature studies have reported that the Middle East has proven oil reserves (47.7%) with over 70% of reservoirs present within these regions exhibiting carbonate lithology [39,126]. Reservoirs within the Kingdom of Saudi Arabia (KSA) are known for exhibiting extreme (harsh) properties, such as high salinity, high temperature, and oil to mixed wet matrix properties with geological layers that are mostly heterogeneous having complex porosity systems. They also contain high permeability channels, which leads them to be characterized with reservoirs with early water breakthrough [13]. Just like other oil and gas reservoirs, water production contributes negatively to overall produced oil and gas. There is a need to overcome these challenges in order to produce the untapped hydrocarbon resources. With the application and use of water-soluble polymers, most publications have shown that these problems can be overturned.

To this effect, some studies have examined the effectiveness of using either a synthetic (HPAM) or biopolymer (XG) in these regions. They found that, though significant success was recorded, some polymers become easily degraded or precipitate at elevated temperature and salinity. As a result, the studies show that difficulty during production becomes imminent. To strike a balance, specific studies were conducted using HPAM based on its usability in other fields around the world, where it was later reported that the degree of hydrolysis leads to an increase in polyacrylic acid attached to the backbone, which increases the sensitivity to hardness and is the key factor causing precipitation at high salinity [22,127,128,129,130]. This information has become an important avenue to explore other polymeric materials.

XG, which is more stable and less sensitive to the above factors (high salinity and high temperature), has also been studied [22,37], where, although it is reportedly biodegradable (forms little amount of precipitates) by nature, the required recovery was achieved as compared to HPAM. This led to greater preference for XG usage compared to HPAM, where some studies have alluded that biodegradability depends on location, well spacing and depth, and the age of the field to which it was applied on. For the future application of XG in these regions (KSA to be specific), we have highlighted a summary of the key screening conditions based on our literature survey in Table 7.

## 6. Summary of Comparison

**Figure 21:**
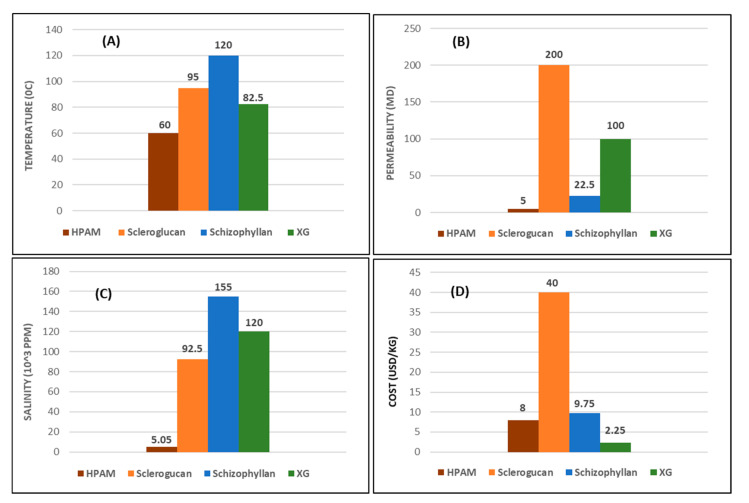
Graphical representation of the averaged polymers’ (**A**) temperature; (**B**) permeability; (**C**) salinity tolerance; (**D**) cost.

This work serves as a complementary review article to some recent publications by [16,22,32,131,132]. Based on the report presented by [22] and other modifications to obtain salinity tolerance [15,39,78,133,134], we have summarized, in detail, the concise chemistry conditions behind the application of these polymeric materials. Table 8 shows the different ranking profiles of these polymers based on their merits and demerits. A critical observation of Table 8 indicates that although biopolymers are known for having good resistance to temperature, as the conclusion obtained from [73] confirms, XG cannot be used beyond 75 to 80 °C after predicting a half-life for 5 years in a moderate to high salinity environment. However, the range of temperature values (75 to 90 °C) presented herein is based on other studies reviewed, as it was demonstrated during the discussion. Figure 21 is a graphical representation (based on averages) of temperature and permeability limits, cost, and tolerance level for salinity of the respective polymers. These further form the basis for the need to study the effects of HPAM and XG spanning through both physical and chemical properties as covered in this article, where they are the most commonly used synthetic and biopolymers, respectively.

## 7. Conclusions

We have presented a comparative study within the confinement of HPAM and XG polymer application for the CEOR process. Background knowledge obtained from this work shows that polymer flooding is a generic and well-established method for both mobility control and effective sweep efficiency in field applications. However, the success of this type of technology in the future depends on the effective development of a fit-for-purpose polymeric system that is cost-effective and environmentally friendly.

This study shows that both polymers exhibit relatively similar rheological behaviours (shear thinning). The principle of decrease in injected fluid mobility for HPAM is based on its molecular weight, which in turn reduces rock permeability, whereas XG reduces injected fluid mobility by increasing its viscosity with little decrease in the reservoir (rock) permeability. In addition to this, HPAM was widely applied previously because of its availability and customizable properties (degree of hydrolysis, molecular weight, etc.) and low cost of manufacture. However, attention is currently shifting towards biopolymers because HPAM is highly susceptible to the conditions of a reservoir (temperature, salinity, shear, etc.), which affects the thickening ability of the synthetic polymer.

In contrast, the XG biopolymer presents a high tolerance ability to salt and temperature because of its distinct helical structure and rigidity. It also has better shear-thinning strength (low power index) with low retention time and resistance factor compared to HPAM. It is presently cheaper and produces less of an environmental impact on society. In the Kingdom of Saudi Arabia, XG and other related biopolymers have been recommended for polymer flooding based on the screening criteria.

**Author Contribution:** The authors are equally involved in the conceptualization, original draft preparation, writing, editing, and reviewing. They have read and agreed to the published version of the manuscript. All authors have read and agreed to the published version of the manuscript.

## Figures and Tables

**Table 1:** Select reported literature on temperature and salinity for XG and HPAM polymers.

Polymer Type	Polymer Concentration (ppm)	Temperature(°C)	NaCl Salinity (ppm)	IPV (PV/Year)	Reference
XG	500–3000	25–65	3000	NC	[13]
XG and Scleroglucan	3000	90–120	1500	NC	[55]
HPAM	1000–2000	NC	3000–7000	0.14–0.20	[70]
HPAM	1500	55–90	NC	NC	[5]
XG	1000–5000	68–70	NC	NC	[35]
XG	400–500	NC	5000	2	[71]
HPAM	1000–2500	30	40,000	0.16–1.8	[72]
HPAM	500–2500	22	20,000	NC	[54]
HPAM	800	<93.3	<100	0.75	[65]
XG	1500	75–80	3000–30,000	NC	[73]
HPAM	1000	100–160	3000–30,000	0.5–1	[60]
PAM	500–5000	46–105	1230	NC	[74]
HPAM	10,000	25	NC	1.2	[57]
HMSPAM	7000	25	NC	1.4	[57]
XG	4000	25	NC	1.2	[57]
XG	300–5000	150	600 **	NC	[63]
HPAM	5000	20–30	5000	0.034–1	[64]
HPAM	1500	25–85	30,000	0.6	[75]
XG	1800	25–85	30,000	0.6	[75]
HPAM	1500	50	15,000	NC	[76]
XG	250–2000	20–120	NC	NC	[77]
NVP-HPAM	1000–4000	120	167,000	NC	[14]
XG	5000	23–77	20,000	NC	[78]

** (NaCl and CaCl_2_), NC = Not Captured.

**Table 2:** Screening criteria for polymer flooding.

PROPERTY	VALUES
Gravity	>25 °API
Viscosity	<150 cP
Composition	Not critical
Oil Saturation	>10% PV
Type of Formation	Sandstone preferred but can be used in carbonates
Net Thickness	Not critical
Average Permeability	>15 mD
Depth	<9000 ft
Temperature	<200 °F
Salinity	<100,000 ppm

**Table 3:** Summarized range of laboratory (experimental) conditions for lab-scale polymer application.

Parameters	XG	HPAM	Other Associated (OA)
MW of polymer (g/mol)	1 × 10^6^–20 × 10^6^	1 × 10^6^–25 × 10^6^	1.3 × 10^6^–20 × 10^6^
Concentration of polymer (ppm)	30–2000	50–10,000	500–3000
Brine salinity (ppm)	661–350,000	250–133,480	5000–186,000
Viscosity of oil (cP)	8–129	1.7–5500	140–18,700
Porosity (%)	10–48	10–45	21–40
Permeability (mD)	18–6000	2.5–13,000	30–12,600
Temperature (°C)	20–100	22–120	22–93

**Table 4:** Summarized range of conditions for pilot-scale polymer application.

Parameters	Range
MW of polymer (g/mol)	5 × 10^6^–37 × 10^6^
Polymer concentration (ppm)	200–2500
Polymer viscosity (cP)	1.35–40
Brine salinity (ppm)	500–120,000
Viscosity of oil (cP)	0.2–10,000
Porosity (%)	11–34
Permeability (mD)	3.9–15,000
Temperature (°C)	22–90

**Table 5:** Summary conditions for effective XG and HPAM field applications. This data set was obtained from the study by [64,65] with additional modification from [32].

PARAMETERS	RANGE
Polymer type	HPAMs and XG
Porosity (%)	4–38
Permeability (mD)	>50
Temperature (°C)	<90
Formation brine salinity (ppm)	<50,000
Divalent ion concentrations (ppm)	<100
Lithology	Sandstone preferred but can be used in carbonates
Water cut for the reservoir to initiate polymer flood (%)	≥95
Clay content level in the reservoir rock	Relatively low
Oil viscosity (cP)	<5000
Oil saturation (%)	>22
Aquifer type	None to weak
Gas cap type	None to weak
The pattern for injection well during the completion	Large hole diameter, high density, and deep penetration
MW of polymer (g/mol) with respective permeability (mD)	For 12 × 10^6^–16 × 10^6^ *k* ≥ 100 For 17 × 10^6^–25 × 10^6^ *k* ≥ 100
Polymer injection rate (PV/year) with respective well spacing (m)	For 0.14–0.16 PV/Yr, spacing = 250 m For 0.14–0.20 PV/Yr, spacing = (150–176) m
Addition of oxygen scavengers to polymer solutions	Alcohol, thiourea, sodium sulphite, and tri-or pentachlorophenol
Addition of biocides to control biological degradation	Formaldehyde

**Table 6:** Key information of some of the field projects reported in the literature.

Field Name/Segment	Polymer Type	Lith.	Temp. (°C)	Formation Water TDS (ppm)	Well Spacing(m)	Polymer Injection Viscosity (cP)	Oil Viscosity (cP)	Recovery Method	Reference
Niagara	PAM	Sand	NR	NR	30	1.35	16	Sec.	[110]
West Cat Canyon	HPAM	Sand	63	NR	200–400	12 (12 rpm at 24 °C)	110	Sec.	[111]
Vernon/Upper Squirrel	HPAM	Sand	24	NR	90–240	1.4	75	Tert.	[112]
Huntington Beach/Garfield	HPAM	Sand	52	31,801	300	NR	76	Tert.	[113]
East Coalinga	XG and Kelzan	Sand	38	NR	150	NR	25 (res.)	Tert.	[109]
Vorhop-Knesebeck/Dogger	Xanthan (state oil)	Sand	56	220,000	NR	4	4	Tert.	[109]
Daqing/Putuahoa form, PO	HPAM (10 MDa)	Sand	45	60,000	100	NR	9.5	Tert.	[114]
Eddesse-Nord	Xanthan	Sand	22	120,000	60	12	7	Tert.	[115]
Chateaurenard/Courtnay	HPAM	Sand	30	400	400	10	40 @ 30 °C	Tert.	[116]
West Castle/Reservoir AQ	HPAM (NaCl 3857)	Sand	NR	NR	66	4	NR	NR	[117]
Daqing/Saertu form	PAM (10 MDa, 22–25% hydro)	Sand	45	7000	200	28	9.5	Tert.	[109]
Captain	HPAM (30% hydro)	Sand	31	13,000–18,000	NR	4.5 (500 ppm)	88 @ 31°C	NR	[109]
Pelican Lake/Wabiskaw	HPAM (13.6 MDa, 32% hydro)	Sand	23	6853	150	30	600–1000	Tert.	[118]
Shuang He/Layer II	HPAM (14.3 MDa, 23% hydro)	Sand	72	4356	250	58 (well head)	7.8	Tert.	[119]
Carmopolis	HPAM (Flopam SNF)	Sand	50	17,091	NR	30	50	Tert.	[120]
Daqing/ZQXB	HPAM	Sand	65	NR	NR	200–250	9.5	Tert.	[121]
Sanand/KS-III	HPAM	Sand	85	NR	200	NR	20	Tert.	[122]
East Bodo/Lloydminster	HPAM (F3630)	Sand	NR	29,000	NR	60	600–2000	Tert.	[123]
Dalia, Camelia	HPAM (18–20 MDa)	Sand	45–56	117,700	1000–1500	3.3	1–11	NR	[124]
Tambaredjo	HPAM	Sand	38	5000	100–600	45 (well head)	300–600	Tert.	[125]

NR = Not recorded; Lith. (lithology); Temp. (temperature); Sec. (secondary); Tert. (tertiary).

**Table 7:** General oil reservoir conditions for Saudi Arabia.

Reservoir Property	Saudi Arabia	
	Northern Area	Southern Area
Depth (ft)	4100–6800	5200–8000
Lithology	Sandstone	Carbonate
Thickness (ft)	20–200	100–300
Porosity (%)	20–29	14–22
Permeability (mD)	1000–3000	100–500
Oil Gravity (API)	27–34	34–37

**Table 8:** Ranking of polymers based on merit and demerits. Some of the data presented were obtained from the study by [22] with additional modifications.

Polymer Chemistry	Polymer Family	Synthetic	Biopolymer		
	Polymer Type	HPAM	Scleroglucan	Schizophyllan	Xanthan Gum
Range of application	Highest temperature limit (°C)	60	95	120	75–90
	Lowest permeability limit (mD)	5	200	150–300	100
Pros and cons involved	$\mathrm{Salinity}\;\mathrm{tolerance}\;\left(\mathrm{ppm}\;\times 10^{3}\right)$	0.1–10	20–165	30–280	20–220
	Tolerance for shear resistance	×××	√√√	√√√	√√
	Tolerance to biodegradation	√√√	×××	×××	××
	Industrial availability	√√√	×	×	√√
	Cost economics (USD/kg)	5–11	30–50	8.5–11	1.5–3
	Logistics and handling	√√	××	××	√√
Application cases	Lab (Experiment)	√√√	×	√	√√√
	Pilot	√√√	√√	√√	√√√
	Field	√√√	×××	×××	√√√

($\left(\surd \surd \surd \right)$) = Excellent, ($\left(\surd \surd \right)$) = better, ($\left(\surd \right)$) = good, (×) = poor, (××) = poorer, and (×××) = poorest.
